# Analysis of Slag-Containing Steamed Concrete’s Composition Efficiency

**DOI:** 10.3390/ma17061300

**Published:** 2024-03-11

**Authors:** Leonid Dvorkin, Vadim Zhitkovsky, Ruslan Makarenko, Yuri Ribakov

**Affiliations:** 1 Institute of Civil Engineering and Architecture, National University of Water and Environmental Engineering, 33028 Rivne, Ukraine; l.i.dvorkin@nuwm.edu.ua (L.D.); v.v.zhitkovsky@nuwm.edu.ua (V.Z.); r.m.makarenko@nuwm.edu.ua (R.M.); 2 Department of Civil Engineering, Ariel University, Ariel 40700, Israel

**Keywords:** thermal power plant slag, concrete, steaming, experimental–statistical model, efficiency criteria, optimal concrete composition

## Abstract

Thermal power plant slag is a waste that is presently obtained from many power stations all over the world. A possible method for its utilization is using it to produce concrete. This paper analyses the effect of thermal power plant slag on the technological properties of concrete mixtures and the mechanical properties of concrete subjected to heat–moisture processing. Quantitative estimates of the investigated factors’ influence on the concrete mixture’s water demand and the strength of steamed concrete were obtained. The influences of TPP slag content and its water demand on concrete composition features as well as concrete strength are shown. The novelty of the work lies in the use of an experimental–statistical model to optimize the composition of steamed concrete using slag from the viewpoint of maximum strength per kilogram of cement. It has been demonstrated that the optimal part of slag in aggregate, which provides maximum strength at 4 h and 28 days after steaming, is 0.5–0.55 and 0.45–0.55, respectively. A method for the design of concrete composition using slag from thermal power plants is proposed.

## 1. Introduction

When solid fuels are burned in the furnaces of thermal power plants (TPPs), ash in the form of dust-like residues, slag, and ash–slag mixtures are formed. They are products of high-temperature (1200–1700 °C) processing of the fuel mineral part [1]. Fly ash has been widely used as an active mineral additive to cement and concrete for many years [2]. Depending on the type of furnace, ash output is from 20 to 40% of the total amount of TPP waste. Other components include ash-slag mixtures and slags that accumulate in a form of dumps, polluting the environment near the power plants [3].

By grain composition, the TPP slag is usually a mechanical mixture of grains that have a size of 0.14 to 30 mm with separate inclusions of larger particles, so it can be considered as a mixture of fine and coarse concrete aggregates [4]. Therefore, TPP slag in lightweight and normal weight concrete is known as the main aggregate, used to partially replace crushed stone (20–50%) and improve the granulometric composition of sands [5].

It was found that crushed slag, unlike natural crushed stone, practically does not contain flat and needle-like grains, clay, and other harmful impurities; therefore, it provides concrete strength up to 30 MPa [4]. There is an intensive interaction between TPP slag and cement during concrete heat-processing. Therefore, immediately after steaming, the strength of slag-containing concrete is about 80% of the set value; after 28 days, it exceeds the strength of normally hardened concrete by 10–15% [6]. Replacing natural aggregates with fuel slag (especially granulated) can lead to a decrease in cement consumption by improving the aggregate mixture granulometric composition, as well as strengthening the contact zone between slag and cement stone [4]. Despite available research results, showing the possibility of using TPP slag in concrete, the effective use of such material is significantly complicated due to the changes in its characteristics (grain size composition, porosity, water demand, etc.), which significantly affect the concrete properties [5]. Hence, the development of a method for determining the optimal concrete composition, which would provide an opportunity to predict and ensure the properties of concrete using TPP slag, taking into account the characteristics of its components and their content, is relevant.

The concrete composition’s effectiveness is determined by its technical and economic indicators, which characterize the rational use of material and energy resources. The rational concrete compositions must also provide a set of standardized properties, taking into account the manufacturing technology of structures and their operation features. Moreover, concrete composition design should also consider the possibility of using technogenic waste and its effect on the concrete properties [7,8,9,10,11]. The main material resource, the rational use of which characterizes, to a large extent, the concrete composition’s effectiveness, is cement [12,13,14,15,16]. As an integral economic criterion for the concrete composition’s optimality, the total costs of its production with standardized properties can be considered [17]. The integral criterion is directly related to other criteria, determining the effectiveness of the cement used in the concrete mixture and energy resources in the products and structures.

## 2. Criteria for Evaluating Material Consumption in Concrete

To analyze the effectiveness of various types of cement that have different cost, quality, and rational use criteria (K_c.r_) [18], the following equation can be used:

(1) $${К}_{c.r}=\frac{S_{c.r}}{S_{c}+S_{t}}=\frac{CC_{c.r}\cdot C_{r}}{CC_{c}\cdot C+S_{t}},$$

where S_c.r_ and S_c_ are the specific costs of cement used per 1 m^3^ of concrete or for the specified structural element, respectively, for the reference and given technological solutions.

S_t_ is the specific cost of the technological methods aimed at reducing the cement costs without decreasing the concrete quality (using additives, electric or steam heating of the mixture, etc.).

CC_c.r_ and CC_c_ are the costs of reference and applied cements, respectively.

C_r_ and C are the consumptions of conditional reference and comparative cements used to obtain concrete with the specified design requirements.

Criterion K_c.r_ can be used to analyze the effectiveness of cements of different costs and qualities when changing their chemical and mineralogical composition, additive content, activity, normal consistency, etc. K_c.r_, enables us to evaluate the cement use effectiveness in the analysis of technological and design solutions related to the reduction of material consumption in products and structures.

The criterion of rational energy costs (K_r.e_) can be used to determine the relative specific consumption of conventional fuel (thermal energy) for the production of 1 m^3^ of concrete or products, including fuel consumption for cement production [19]:

(2) $${К}_{r.e}=\frac{C_{r}\cdot F_{c.r}+F_{ad}}{C\cdot F_{c}+F_{st}+F_{ad}},$$

where C_r_ and C are consumptions of the conditional reference and actually used cements required to obtain concrete with given design properties, kg/m^3^.

F_c.r_ and F_c_ are the «equivalent coal» (the heat of combustion is 29.3 MJ/kg) consumption for obtaining 1 kg of reference and the actually used cements.

F_st_ is the «equivalent coal» consumption for product steaming.

F_ad_ is the additional fuel consumption for technological purposes.

Criterion K_r_._e_ allows us to compare the energy consumption for the production of concrete with different properties and to evaluate the efficiency of using thermal energy in different compositions of concrete, both under conditions of normal hardening and heat processing. With the help of this criterion, researchers can choose the optimal heat treatment modes based on fuel consumption. When comparing normally curing concrete and steamed concrete, provided that the same type of cement is used, approaching the criterion value of 1 indicates an increase in the energy efficiency of the concrete steaming process. It is also possible to use the criterion to compare the energy efficiency of various technological solutions—adding mineral and chemical admixtures, concrete mixture heating, etc.

Using transition coefficients when determining K_r.e_, it is possible to take into account, along with heat, electricity consumption (1 kWh~0.34 kg of «equivalent coal») [19]. All possible technological solutions that lead to an increase in K_r_._e_ without an unacceptable decrease in line productivity and a significant increase in costs are progressive and can be recommended for implementation.

When using a certain type of cement and certain technological parameters of concrete production (concrete mixture workability and methods and modes of heat processing), the concrete composition’s effectiveness can be characterized using the ratio of the most important standardized parameter to the cement consumption (C). For this purpose, the following relation can be used [17]:

(3) $$L=\frac{f_{cm}}{C},$$

where f_cm_ is the compressive strength of concrete with normal or accelerated hardening at a certain age.

For a more in-depth analysis, it is advisable to consider expression (3), taking into account important technological factors. In particular, for this purpose, it is possible to use the well-known dependences for concrete strength [17,20]:

(4) $$f_{cm}=AR_{c}(C/W-b),$$

(5) $$f_{cm}=AR_{c}(\frac{C+K_{c.e}D}{W}-b),$$

where R_c_ is the cement strength, MPa;

C/W is the cement–water ratio;

D is the mineral additive content, kg/m^3^;

A and b are empirical coefficients that depend on the characteristics of the initial materials and other factors;

K_c.e_ is the coefficient of additive cementing efficiency [17].

The most detailed analysis with an appropriate search for the maximum value of criterion L is achieved when using detailed quantitative dependences of the concrete strength to cement consumption ratio, taking into account the set of influencing technological factors. With this aim can be used experimental-statistical models obtained by experiment design.

## 3. Research Aims, Scope, and Novelty

The aim of the present study is to obtain quantitative dependencies to enable the development of a method for designing optimal compositions of concrete using slag from thermal power plants, whereby a given strength is achieved at 4 h and 28 days after steaming. To achieve this goal, the following tasks should be solved:

- Implementing a planned experiment in order to study the influence of characteristics and the consumption of concrete components, including slag from thermal power plants, on the steamed concrete strength and the optimality criterion L (Equation (3));
- Carrying out statistical processing of experimental data, establishing quantitative estimates of the individual and joint influence of the investigated factors, and obtaining experimental–statistical models of the influence of concrete composition parameters and raw material characteristics;
- Analyzing the obtained models and evaluating the influence of factors on the steamed concrete strength and optimality criterion L as well as to finding the optimal values of the factors;
- Developing a method for designing the optimal composition of steamed concrete using TPP slag with a given strength, while considering criterion L.

To solve the above-mentioned tasks, the method of mathematical experiment planning was used [21,22]. This method is based on carrying out the experiments according to a pre-designed scheme, characterized by optimal properties in terms of the experimental work quantity and statistical requirements. The theory of experiment planning is based on probabilistic statistical methods that enable researcher to theoretically find the minimum required number and composition of experiments, as well as their order, to obtain quantitative relationships between the investigated parameter and the factors influencing it. The main task of mathematical experiment planning is to obtain a mathematical model that characterizes the relationship between the optimization parameter and independent variables (factors) [23].

As a result of statistically processing the experimental data, the regression coefficients of the investigated factors are obtained. Based on the magnitude of the regression coefficients, one can judge the effects—the influence degree of the relevant factors.

The result of mathematical experiment planning in concrete technology is usually presented in the form of an experimental–statistical model, which is a second-order polynomial [23]:

(6) $$y=b_{0}+\sum_{i=1}^{k}b_{i}x_{i}+\sum_{i=1}^{k}b_{ij}x_{i}x_{j}+\sum_{i=1}^{k}b_{ii}x_{1}^{2},$$

where y is the initial parameter;

b_0_, b_i_, b_ij_, and b_ii_ are the regression coefficients;

x_i_, x_ij_, and x_ii_ are the investigated factors;

k is the number of factors.

Optimizing the composition of concrete using TPP slag is aimed at finding the consumption of concrete components at which the specified strength parameters will be achieved and the maximum values of the optimization criterion L will be ensured (Equation (3)).

The main novelty of this study is that the proposed approach allows us to find a concrete composition that ensures the calculated strength characteristics at maximum cement use efficiency (criterion L), taking into account the characteristics of TPP slag and other aggregates.

## 4. Materials and Research Methods

To obtain the concrete mixture, Portland cement CEM 32.5 was used. The cement’s physical and mechanical properties are given in Table 1.

As a fine aggregate, quartz sand was use, with partial or complete replacement by fuel slag; crushed granite stone [20] served as a coarse aggregate. The water demand was used as the main aggregate characteristic [24], which was determined to fall within the accepted variation-in-factors range during the experiment, depending on the size, porosity, and content of impurities [25] (Table 2).

Slag from the ash–slag dumps of Burshtynskaya TPP (Ukraine) was used. It had the following chemical composition, %: SiO_2_—48.6; Al_2_O_3_—23.3; Fe_2_O_3_—15.3; CaO—4.4; MgO—2.2; Na_2_O—0.5; K_2_O—1.6; and SO_3_—0.47.

According to the known methodology [24], the water demands of sand (W_s_), slag (W_sl_), and crushed stone (W_sc_) were found using the following expressions:

(7) $$W_{s(sl)}=\frac{{\left(W/C\right)}_{m}-{\left(W/C\right)}_{p}}{2}\cdot 100\%,$$

(8) $$W_{sc}=\frac{{\left(W/C\right)}_{c}-{\left(W/C\right)}_{m}}{3.5}\cdot 100\%,$$

where (W/C)_p_ is the W/C of the cement paste with cone spreading on a Flow Table [26] of about 170 mm, which approximately corresponds to its normal consistency.

(W/C)_m_ is the W/C of the mortar on the tested sand or slag, at which it has the same cone slump as the cement paste of normal consistency.

(W/C)_c_ is the W/C of the concrete mixture at which the same workability as that of the mortar mixture at (W/C)_m_ is achieved.

To find the water demand of sand, a cement–sand mortar with a cement–sand ratio of 1:2 (by weight) and a concrete mix with a cement–sand–crushed stone ratio of 1:2:3.5 (by weight) were prepared. The workability of the mortar corresponded to the cone spreading on a Flow Table [26] of 170 mm. The slump of the concrete mixtures [27] was 160 mm. The water demand was calculated according to Equations (7) and (8).

The concrete mixes corresponded to the S4 class with a slump range of 160–210 mm [27]. In order to reduce the water content, an additive of naphthalene–formaldehyde superplasticizer in the amount of 0.7% of the cement weight was added to all of the investigated concrete mixture compositions.

Along with the water demands of sand, slag, and crushed stone, the influences of the cement–water ratio, the crushed stone part in the aggregate mixture, and the part of slag in the fine aggregate on the concrete compressive strength were also studied. The strength of concrete sample was studied after steaming at 80 °C using the 2 + 3 + 5 + 2 h mode (holding before steaming + temperature rise + holding at maximum temperature + cooling).

To find optimal concrete compositions, experiments were performed using the mathematical planning method according to the 3-level, 6-factor Box–Behnken plan [21,23] (Table 3 and Appendix A, Table A1). This plan includes 54 experimental points and enables researchers to study the nonlinear influence of six factors using a minimum number of experiments. The plan was selected using the Statistica 6.0 (Statsoft) software package [28]. Overall, 54 series of 100 mm × 100 mm × 100 mm cubic specimens were produced. Each series consisted of 6 specimens, 3 of which were tested 4 h after steaming and the other 3 after 28 days of curing. The specimens were manufactured and tested in accordance with the requirements of modern normative documents [29].

## 5. Experimental Data and Its Processing

The experiment planning conditions are given in Table 3, the planning matrix is presented in Appendix A, and Table A1 and the experimental data are shown in Appendix A, Table A2. After statistical processing [21,23,28] of the experimental results (Table A2), a complex of adequate experimental–statistical models with a confidence probability of 95% was obtained in the form of polynomial equations (Equation (6)), which are given in Table 4. The significance of the coefficients of the equations was checked using Student’s test [23]. The adequacy of the equations was checked by calculating the adequacy variance and using Fisher’s criterion. The calculations were performed using the Statistica 6.0 (Statsoft) software package [28].

**Table 4:** Experimental–statistical models of the slag-containing concrete properties.

Properties	Equation
Water demand of the concrete mixture, L/m^3^	W = 208.6 − 7.7X_1_ − 3.4X_2_ − 5.5X_3_ + 8.4X_4_ + 2.5X_5_ + 10.0X_6_ + 17.0X_1_^2^ + 26.5X_2_^2^ + 1.5X_3_^2^ − 1.0X_4_^2^ + 12.1X_1_X_2_ + 5.6X_1_X_3_ − 5.1X_1_X_5_ + 8.5X_2_X_4_ − 10X_2_X_5_ (9)
Concrete compressive strength, 4 h after steaming, MPa	f_cm4h_ = 18.9 + 2.1X1 + 1.0X_2_ − 0.4X_3_ + 0.5X_4_ − 0.5X_5_ + 8.9X_6_ − 0.8X_1_^2^ − 2.0X_2_^2^ + 0.3X_3_^2^ − 0.7X_4_^2^ + 1.1X_5_^2^ + 1.5X_6_^2^ − 1.3X_1_X_3_ − 0.3X_1_X_5_ + 0.7X_1_X_6_ − 0.3X_2_X_3_ + 0.3X_2_X4 + 0.2X_2_X_5_ + 0.7X_2_X_6_ − 0.9X_3_X_4_ − 0.3X_3_X_5_ − 0.8X_4_X_5_ (10)
Concrete compressive strength, 28 days after steaming, MPa	f_cm28d_ = 30.9 + 2.6X_1_ + 2.5X_2_ + 1.3X_3_ + 1.8X_4_ + 1.8X_5_ + 13X_6_ + 0.4X_1_^2^ − 1.5X_2_^2^ + 1.3X_3_^2^ − 1.0X_4_^2^ + 0.9X_5_^2^ − 0.8X_6_^2^ − 1.4X_1_X_2_ − 0.6X_1_X_3_ − 0.8X_1_X_4_ − 0.8X_1_X_5_ + 2.0X_1_X_6_ + 1.1X_2_X_3_ − 1.3X_2_X_5_ + 2.4X_2_X_6_ − 0.3X_3_X_4_ − 0.4X_3_X_5_ − 0.3X_4_X_5_ (11)
Criterion of cement efficiency for concrete, 4 h after steaming (L_4h_), MPa/kg	L_4h_ = 0.048 + 0.006X_1_ + 0.002X_2_ − 0.002X_3_ − 0.003X_4_ + 0.005X_6_ − 0.005X_1_^2^ − 0.009X_2_^2^ − 0.001X_4_^2^ + 0.002X_5_^2^ − 0.001X_6_^2^ − 0.001X_1_X_2_ − 0.003X_1_X_3_ − 0.010X_1_X_5_ + 0.002X_2_X_5_ + 0.001X_2_X_6_ − 0.002X_3_X_4_ − 0.015X_3_X_5_ − 0.002X_4_X_5_ (12)
Criterion of cement efficiency for concrete, 28 days after steaming (L_28d_), MPa/kg	L_28d_ = 0.078 + 0.008X_1_ + 0.006X_2_ + 0.005X_3_ − 0.007X_4_ − 0.005X_5_ + 0.006X_6_ − 0.004X_1_^2^ − 0.011X_2_^2^ + 0.002X_3_^2^ − 0.002X_4_^2^ + 0.001X_5_^2^ − 0.005X_6_^2^ − 0.006X_1_X_2_ − 0.002X_1_X_4_ + 0.001X_1_X_5_ + 0.003X_1_X_6_ + 0.002X_2_X_3_ − 0.002X_2_X_4_ + 0.004X_2_X_6_ + 0.001X_3_X_6_ + 0.002X_4_X_6_ + 0.002X_5_X_6_ (13)

The obtained mathematical models (Table 4) enabled us to analyze the influence of the investigated factors on the properties of concrete with thermal power plant slag.

**Figure 1:**
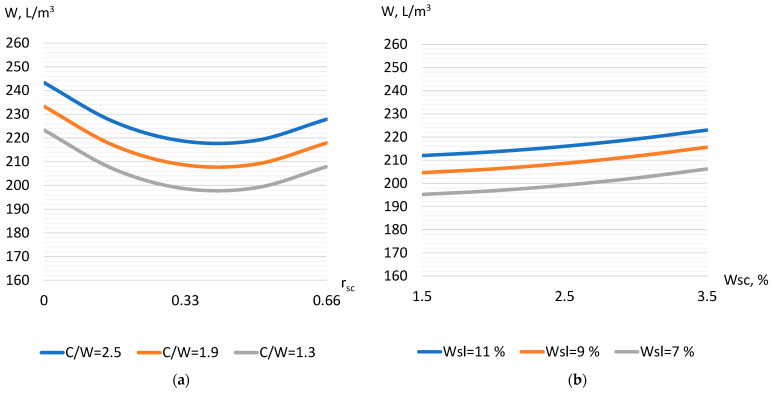
The influence of the crushed stone part, C/W (**a**) and water demand of sand and slag (**b**) on the water demand of the concrete mixture with thermal power plant slag.

The concrete mixture water demand (Equation (9)) varied quite widely: from 185 to 275 L/m^3^. Such a wide range of change in water demand is caused by fluctuations in the concrete composition within rather wide limits, as well as by changes in the types of aggregates and their properties, since aggregates with different water demand were used, such as thermal power plant slag, crushed stone, and sand. Factors X_1_ and X_2_, characterizing the part of crushed stone in the aggregate, and the part of slag in sand cause a decrease in water demand when they increase. The maximum reduction in water demand causes an increase in the part of crushed stone in concrete (X_1_), which is logically explained by an increase in the total size of the aggregate (Figure 1a). The influence of slag (X_2_) in this case is somewhat lower. Factors X_3_–X_6_, when they increase, cause an increase in the concrete mixture water demand. The maximum increase in water demand is observed with an increase in the cement–water ratio (X_6_). This is mostly caused by an increase in the concrete mixture viscosity as a result of an increase in the amount of cement paste in this experiment within rather wide limits. Since factors X_3_–X_5_ characterize the water demand of aggregates (crushed stone, slag, and sand), the increase in the value of these indicators is reflected, accordingly, in the total water demand of the concrete mixture (Figure 1b). Since power plant slag, in most cases, acts as an alternative aggregate in concrete [30], it should be expected that a change in the water demand of a concrete mixture, due to the water demand of aggregates, will largely determine the change in concrete strength.

**Figure 2:**
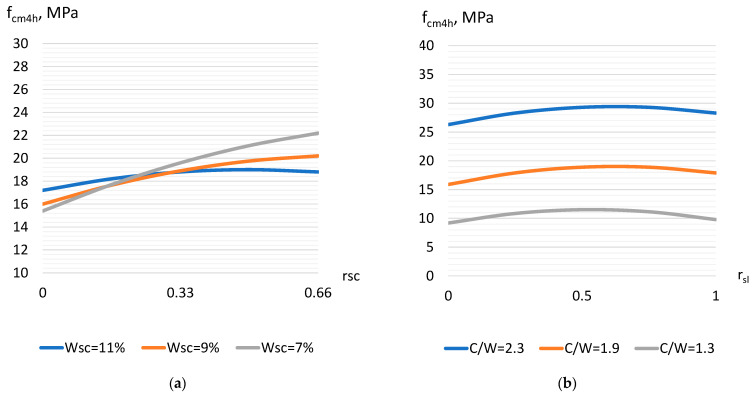
The influence of water demand and crushed stone part (**a**) as well as C/W and slag part (**b**) on concrete strength 4 h after steaming.

The strength of the concrete with TPP slag 4 h after steaming (Equation (10)) varied from 8 to 33 MPa. The most significant factor that causes an increase in strength is the cement–water ratio (X_6_) [17]. An increase in C/W from 1.3 to 2.5 causes an increase in compressive strength by almost 3 times. Additionally, the increase in strength, but to a much lower extent, is caused by an increase in the crushed stone part in the aggregate (factor X_1_) (Figure 2a) and an increase in the part of slag in sand (factor X_2_). Factor X_2_ is characterized by a significant negative quadratic effect, which indicates the existence of a maximum zone in the varied range of its influence. An increase in strength is observed up to values of the slag part of 0.5–0.6 (Figure 2b). Then, the strength begins to decrease, most likely due to the increased voids of the aggregate and increase in concrete porosity [31]. When factors that characterize the aggregates water demand (X_3_–X_5_) increase, it causes a decrease in concrete strength. The most significant, in the model of strength 4 h after steaming, is the interaction of factors X_1_ and X_3_—as the crushed stone water demand increases, its positive effect on strength decreases.

**Figure 3:**
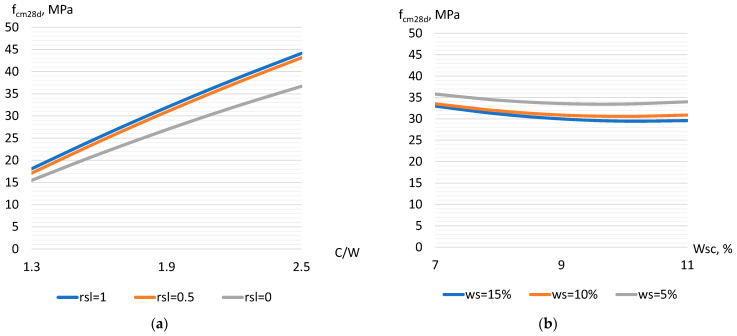
The effect of C/W and the part of slag (**a**) as well as the water demand of slag and sand (**b**) on the strength of steamed concrete after 28 days.

On the 28th day of hardening after steaming (Equation (11)), the described trends of influence of the investigated factors on the strength of the concrete with slag are mostly preserved (Figure 3a,b). The strength value at 28 days is 1.4–1.5 times higher than that at 4 h after steaming (achieved values from 15 to 45 MPa) (Figure 3a).

**Figure 4:**
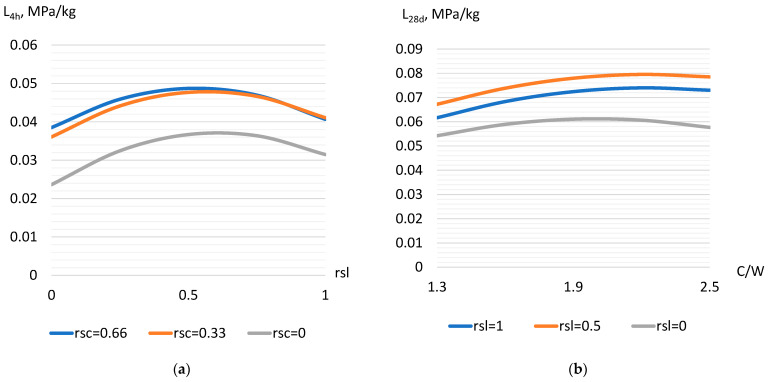
The influence of the investigated factors on the cement use efficiency criterion for strength 4 h after steaming (**a**) and after 28 days (**b**).

Models of criterion L obtained on the basis of strength values after steaming (L_4h_ and L_28d_, Equations (12) and (13)) allow us to establish the optimal composition of concrete with slag, at which the strength achieved per each kilogram of cement is the maximum. According to the strength 4 h after steaming, the L_4h_ criterion varies from 0.022 to 0.058 MPa/kg. After 28 days of hardening, the values of the criterion (L_28d_) are higher—from 0.041 to 0.094 MPa/kg. Analyzing the influence of factors on criteria L_4h_ and L_28d_, it should be noted that factors X_1_ (crushed stone part), X_2_ (slag part) (Figure 4a), and X_6_ (cement–water ratio) (Figure 4b), when they increase, cause an increase in the criterion values. The influence of these factors on the models is also characterized by the presence of a quadratic effect, which indicates the existence of an optimal values region. The presence of an optimum zone for factors X_1_ and X_2_, which characterize an increase in the proportion of crushed stone and slag in concrete, is caused by an increase in voids with a high number of coarse fractions and a simultaneous decrease in the concrete mixture water demand [32]. The optimal zone of the C/W factor (about 2–2.3) (Figure 4b) is most likely caused by a significant increase in the mixture viscosity and the occurrence of some concrete under consolidation with a simultaneous increase in the cement paste density [19,24]. Factors that characterize the increase in aggregate water demand (X_3_–X_5_) cause a decrease in the values of concrete composition efficiency criteria.

The resulting models (Equations (9)–(13)) can be used to predict the properties of concrete with thermal power plant slag and design optimal concrete compositions. When calculating the composition, we first calculate the cement–water ratio under the condition of ensuring concrete strength 4 h after steaming (f_cm4h_, Equation (10)) and after 28 days (f_cm28d_, Equation (11)).

The values of crushed stone and slag parts are calculated according to the equations obtained via the joint solution of models L_4h_ and L_28d_, provided that the maximum value of the cement efficiency coefficient is ensured. The water demand necessary to achieve the optimal concrete mixture consistency is calculated according to Equation (9). The consumptions of sand, crushed stone, and slag are obtained using the following sequence:

r_sl_ = r′_sc_ · r,     r_s_ = r − r_sl_; (14)

where

r = 1 − r_sc_; (15)

CS = r_sc_ · K, Sl = r_sl_ · K,     S = r_s_ · K; (16)

where

K = (1000 − C/ρ_c_ − W/ρ_w)_/(r_sc_/ρ_cs_ + r_sl_/ρ_sl_ + r_s_/ρ_s_). (17)

## 6. Example

It is necessary to determine the optimal composition of slag-containing concrete with a mixture cone slump of 160–200 mm and strength 4 h after steaming f_cm4h_ = 25.0 MPa (concrete class C20/25) for Portland cement CEM 32.5 (ρ_c_ = 3.1 kg/L, NC = 27.3%), quartz sand (W_s_ = 10%, ρ_s_ = 2.69 kg/L), granite crushed stone (W_sc_ = 2.5%, ρ_sc_ = 2.61 kg/L), thermal power plant slag (W_sl_ = 9%, ρ_sl_ = 2.45 kg/L), and naphthalene formaldehyde type superplasticizer in the amount of 0.7% of the cement weight. The approximate value of the crushed stone part in the aggregates should be equal to 0.66—the part of slag in fine aggregates should be 0.5.

According to Equation (10), substituting the necessary value of strength and taking the values of factors X_1_–X_5_ according to the condition, we set the necessary C/W. C/W = 2.32.

**Figure 5:**
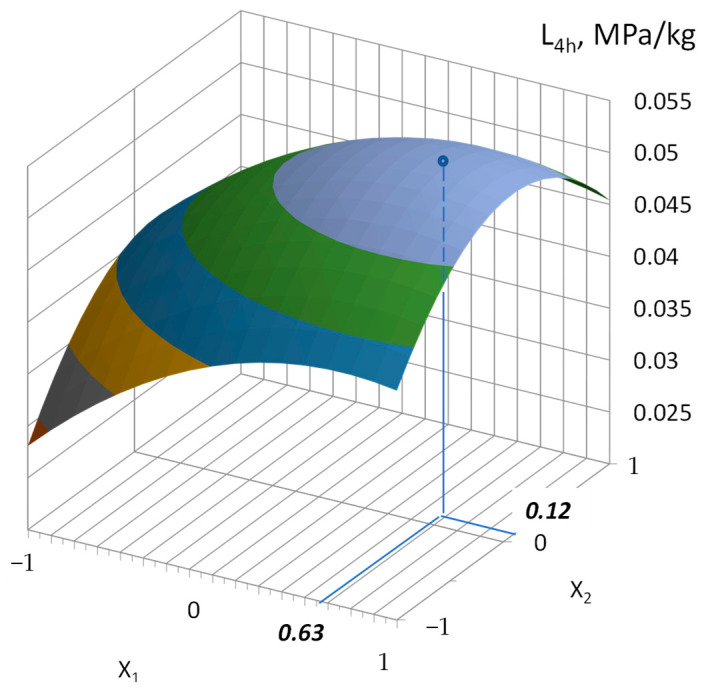
Example of graphic determination of the optimal crushed stone part in the aggregates (X_1_) and the part of slag in the fine aggregates (X_2_) according to the maximum value of the L_4h_ criterion. When constructing the response surface according to Equation (12), the following values were adopted: C/W = 2.32, W_s_ = 10%, W_sc_ = 2.5%, and W_sl_ = 9%.

According to Equation (12), we determined the optimal part of crushed stone in the mixture of aggregates (X_1_) and the part of slag in the fine aggregates (X_2_) with this C/W and the characteristics of the aggregates according to the condition (Figure 5):

X_1_ = 0.63;
X_2_ = 0.12.

In natural units:

r_sc_ = 0.33 · 0.63 + 0.33 = 0.54;
r_sl_ = 0.5 · 0.12 + 0.5 = 0.56.

After that, the water demand was obtained according to Equation (10): W = 218 kg/m^3^.

The cement consumption: C = 218 · 2.32 = 506 kg/m^3^.

The superplasticizer content: SP = 506 · 0.7/100 = 3.54 kg/m^3^.

The estimated values of optimal parts of slag *r_sl_* and sand *r_s_* in the mixture of aggregates according to Equations (14) and (15):

*r_sc_* = 1 − 0.54 = 0.46; *r_sl_* = 0.46 · 0.56 = 0.26; and *r_s_* = 0.46 − 0.26 = 0.2

According to Equations (16) and (17), the consumption of crushed stone, slag and sand are as follows:

K = (1000 − 506/3.1−218/1)/(0.54/2.61 + 0.2/2.69 + 0.26/2.45) = 1597 kg/m^3^;
CS = 1597 · 0.54 = 862 kg/m^3^;
Sl = 1597 · 0.26 = 415 kg/m^3^;
S = 1597· 0.2 = 320 kg/m^3^.

## 7. Conclusions

A planned experiment was carried out using a three-level Box–Behnken plan for six factors, containing fifty-four experimental points. The following factors were selected as the investigated factors: part of crushed stone in the aggregate mixture; part of TPP slag in fine aggregate; and water demand of crushed stone, water demand of slag, water demand of sand, cement–water ratio. The concrete strengths at 4 h and 28 days after steaming were obtained experimentally. The value of the concrete composition efficiency criterion L (strength per kilogram of cement) was calculated.

Using Statistica 6.0 (Statsoft) software, statistical processing of experimental data was carried out. Quantitative estimates of the investigated factors’ influence were established. Finally, experimental–statistical models describing the influence of concrete composition parameters and raw material characteristics on the compressive strength of steamed concrete with TPP slag and on criterion L were obtained.

As a result of the obtained model’s analysis, the influence of TPP slag consumption and aggregate characteristics on the water demand of the concrete mixture and strength of steamed concrete at 4 h and 28 days after steaming was established for a wide range of concrete compositions. It has been shown that the optimal value of the slag part in the aggregate, which allows the maximum strength at 4 h after steaming (from 13.2 to 32.3 MPa (depending on C/W)) to be achieved taking into account the efficiency of concrete composition criterion L, is 0.5–0.55. Maximum strength at 28 days after steaming (from 24.6 to 52.8 MPa) is achieved using a slag part of 0.45–0.55.

A method for selecting the optimal composition of steamed concrete with TPP slag has been developed. It provides maximum strength with minimal cement consumption.

## Figures and Tables

**Table 1:** Physical and mechanical properties of Portland cement.

Normal Consistency, %	Setting Time		Compressive Strength, MPa		
	Initial	Final	2 Days	7 Days	28 Days
27.3	52 min	2 h 35 min	14.5	21.3	46.2

**Table 2:** Water demand of aggregates.

Aggregate	Fineness Modulus	Maximal Fraction, mm	The Content of Dusty and Clay Impurities, %	Water Demand, %
Quartz sand	2.9	5	0.5	5
	1.5	2.5	3.5	10
	1.1	1.25	5.2	15
Thermal power plant slag	2.5	5	0.8	7
	1.8	5	3.9	9
	1.5	2.5	4.5	11
Crushed granite stone	–	20	0.5	1.5
	–	20	2.5	2.5
	–	20	3.5	3.5

**Table 3:** Experiment planning conditions.

Factors		Variation Levels			Variation Interval
Natural	Coded	−1	0	+1	
Part of crushed stone in the aggregate mixture r_sc_	X_1_	0	0.33	0.66	0.33
Part of slag in fine aggregate r_sl_	X_2_	0	0.5	1.0	0.5
Water demand of crushed stone (w_sc_), %	X_3_	1.5	2.5	3.5	1.0
Water demand of slag (w_sl_), %	X_4_	7.0	9.0	11.0	2.0
Water demand of sand (w_s_), %	X_5_	5.0	10.0	15.0	5.0
Cement–water ratio	X_6_	1.3	1.9	2.5	0.6

## Data Availability

Data are contained within the article.

## Appendix A

**Table A1:** Experiment planning matrix.

**No.**	**Coded Factors**					
	**X_1_**	**X_2_**	**X_3_**	**X_4_**	**X_5_**	**X_6_**
1	−1	−1	0	−1	0	0
2	+1	−1	0	−1	0	0
3	−1	+1	0	−1	0	0
4	+1	+1	0	−1	0	0
5	−1	−1	0	+1	0	0
6	+1	−1	0	+1	0	0
7	−1	+1	0	+1	0	0
8	+1	+1	0	+1	0	0
9	0	−1	−1	0	−1	0
10	0	+1	−1	0	−1	0
11	0	−1	+1	0	−1	0
12	0	+1	+1	0	−1	0
13	0	−1	−1	0	+1	0
14	0	+1	−1	0	+1	0
15	0	−1	+1	0	+1	0
16	0	+1	+1	0	+1	0
17	0	0	−1	−1	0	−1
18	0	0	+1	−1	0	−1
19	0	0	−1	+1	0	−1
20	0	0	+1	+1	0	−1
21	0	0	−1	−1	0	+1
22	0	0	+1	−1	0	+1
23	0	0	−1	+1	0	+1
24	0	0	+1	+1	0	+1
25	−1	0	0	−1	−1	0
26	+1	0	0	−1	−1	0
27	−1	0	0	+1	−1	0
28	+1	0	0	+1	−1	0
29	−1	0	0	−1	+1	0
30	+1	0	0	−1	+1	0
31	−1	0	0	+1	+1	0
32	+1	0	0	+1	+1	0
33	0	−1	0	0	−1	−1
34	0	+1	0	0	−1	−1
35	0	−1	0	0	+1	−1
36	0	+1	0	0	+1	−1
37	0	−1	0	0	−1	+1
38	0	+1	0	0	−1	+1
39	0	−1	0	0	+1	+1
40	0	+1	0	0	+1	+1
41	−1	0	−1	0	0	−1
42	+1	0	−1	0	0	−1
43	−1	0	+1	0	0	−1
44	+1	0	+1	0	0	−1
45	−1	0	−1	0	0	+1
46	+1	0	−1	0	0	+1
47	−1	0	+1	0	0	+1
48	+1	0	+1	0	0	+1
49	0	0	0	0	0	0
50	0	0	0	0	0	0
51	0	0	0	0	0	0
52	0	0	0	0	0	0
53	0	0	0	0	0	0
54	0	0	0	0	0	0

**Table A2:** Experimental data.

**No.**	**Cement Consumption, C, kg/m^3^**	**Water Consumption, W, L/m^3^**	**Concrete Compressive Strength, MPa** **(Average of Three Tested Specimens)**		**Criterion L, MPa/kg** **(Values Calculated According to Equation (3))**	
			**4 h after Steaming (f_cm4h_)**	**28 Days after Steaming (f_cm28d_)**	**4 h after Steaming (L_4h_)**	**28 Days after Steaming (L_28d_)**
1	521	274	13.1	23.3	0.025	0.045
2	446	235	17.3	32.9	0.039	0.074
3	430	226	14.5	31.1	0.034	0.072
4	447	235	18.7	35.1	0.042	0.079
5	521	274	11.5	21.3	0.022	0.041
6	446	235	15.7	27.7	0.035	0.062
7	494	260	14.1	29.1	0.029	0.059
8	511	269	18.3	29.9	0.036	0.059
9	422	222	17.8	31.6	0.042	0.075
10	447	235	20	37	0.045	0.083
11	443	233	18.2	27.6	0.041	0.062
12	468	246	19.2	37.4	0.041	0.080
13	469	247	17	31.4	0.036	0.067
14	418	220	20	31.6	0.048	0.076
15	490	258	16.2	25.8	0.033	0.053
16	439	231	18	30.4	0.041	0.069
17	241	185	11.1	20.2	0.046	0.084
18	255	196	12.1	18.2	0.047	0.071
19	263	202	11.9	17.2	0.045	0.065
20	277	213	9.3	14	0.034	0.051
21	513	205	28.9	46.2	0.056	0.090
22	541	216	29.9	44.2	0.055	0.082
23	555	222	29.7	43.2	0.054	0.078
24	583	233	27.1	40	0.047	0.069
25	411	216	16.3	31.1	0.040	0.076
26	401	211	21.1	37.9	0.053	0.094
27	443	233	16.9	29.7	0.038	0.067
28	433	228	21.7	33.3	0.050	0.077
29	440	232	17.5	28.1	0.040	0.064
30	391	206	21.1	34.9	0.054	0.089
31	472	248	14.9	25.5	0.032	0.054
32	423	223	18.5	29.1	0.044	0.069
33	281	216	11	16.9	0.039	0.060
34	298	229	11.2	19.7	0.038	0.066
35	313	241	9.6	15.9	0.031	0.051
36	278	214	10.6	13.5	0.038	0.048
37	590	236	27.4	38.1	0.046	0.065
38	623	249	30.4	50.5	0.049	0.081
39	653	261	26	37.1	0.040	0.057
40	586	234	29.8	44.3	0.051	0.076
41	278	214	8.7	18.9	0.031	0.068
42	272	210	14.1	21.3	0.052	0.078
43	307	236	10.5	17.5	0.034	0.057
44	272	209	10.7	17.5	0.039	0.064
45	584	234	25.1	40.9	0.043	0.070
46	574	230	33.3	51.3	0.058	0.089
47	640	256	26.9	39.5	0.042	0.062
48	573	229	29.9	47.5	0.052	0.083
49	396	209	18.4	30.9	0.046	0.078
50	396	210	18.9	30.9	0.048	0.077
51	396	210	19	30.9	0.048	0.077
52	396	208	19.2	30.9	0.048	0.079
53	396	211	18.7	30.9	0.047	0.079
54	396	208	18.9	30.9	0.048	0.076
